# Supplementary material for: The Nitro Group Reshapes the Effects of Pyrido[3,4-g]quinazoline Derivatives on DYRK/CLK Activity and RNA Splicing in Glioblastoma Cells
Source: Cancers (Basel). 2024 Feb 19;16(4):834. doi: 10.3390/cancers16040834 (PMC10886777; doi:10.3390/cancers16040834)
Supplement: Supplementary file 1 [file cancers-16-00834-s001.zip › Supplementary Materials Figures S1-S8 and Tables S1 and S2.pdf]

Supplementary Materials

The Nitro Group Reshapes the Effects of Pyrido[3,4-*g*]quinazoline Derivatives on DYRK/CLK Activity and RNA Splicing in Glioblastoma Cells

Sophia S. Borisevich, Tatyana E.Aksinina, Margarita G. Ilyina, Victoria O. Shender, Ksenia S. Anufrieva, Georgij P. Arapidi, Nadezhda V. Antipova, Fabrice Anizon, Yannick J. Esvan, Francis Giraud, Victor V. Tatarskiy, Pascale Moreau, Mikhail I. Shakhparonov, Marat S. Pavlyukov and Alexander A. Shtil

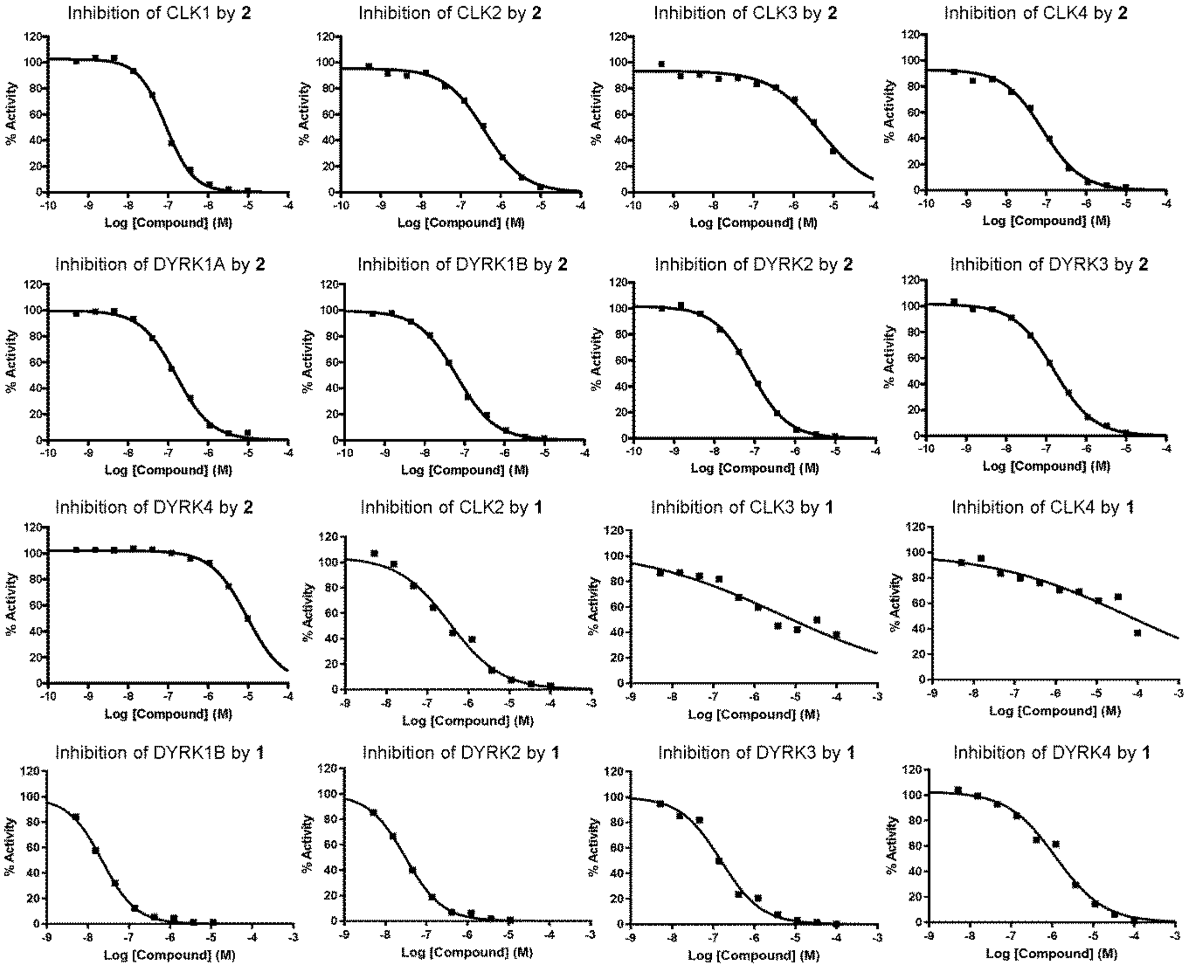

Figure S1. Log dose response analysis of the effects of compounds **1** and **2** on the kinase activity of DYRK/CLK family members.

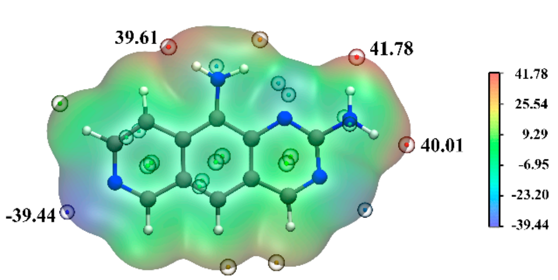

1

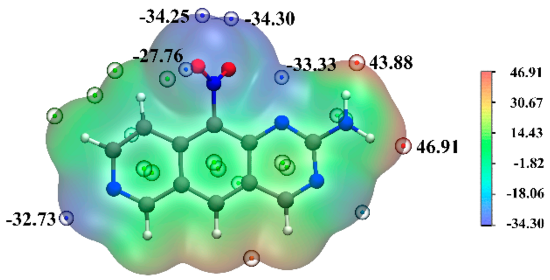

2

Figure S2. ESP mapping. Local ESP minimums and maximums (kcal/mol) on van der Waals isosurfaces of **1** and **2** are shown as red and blue spheres, respectively.

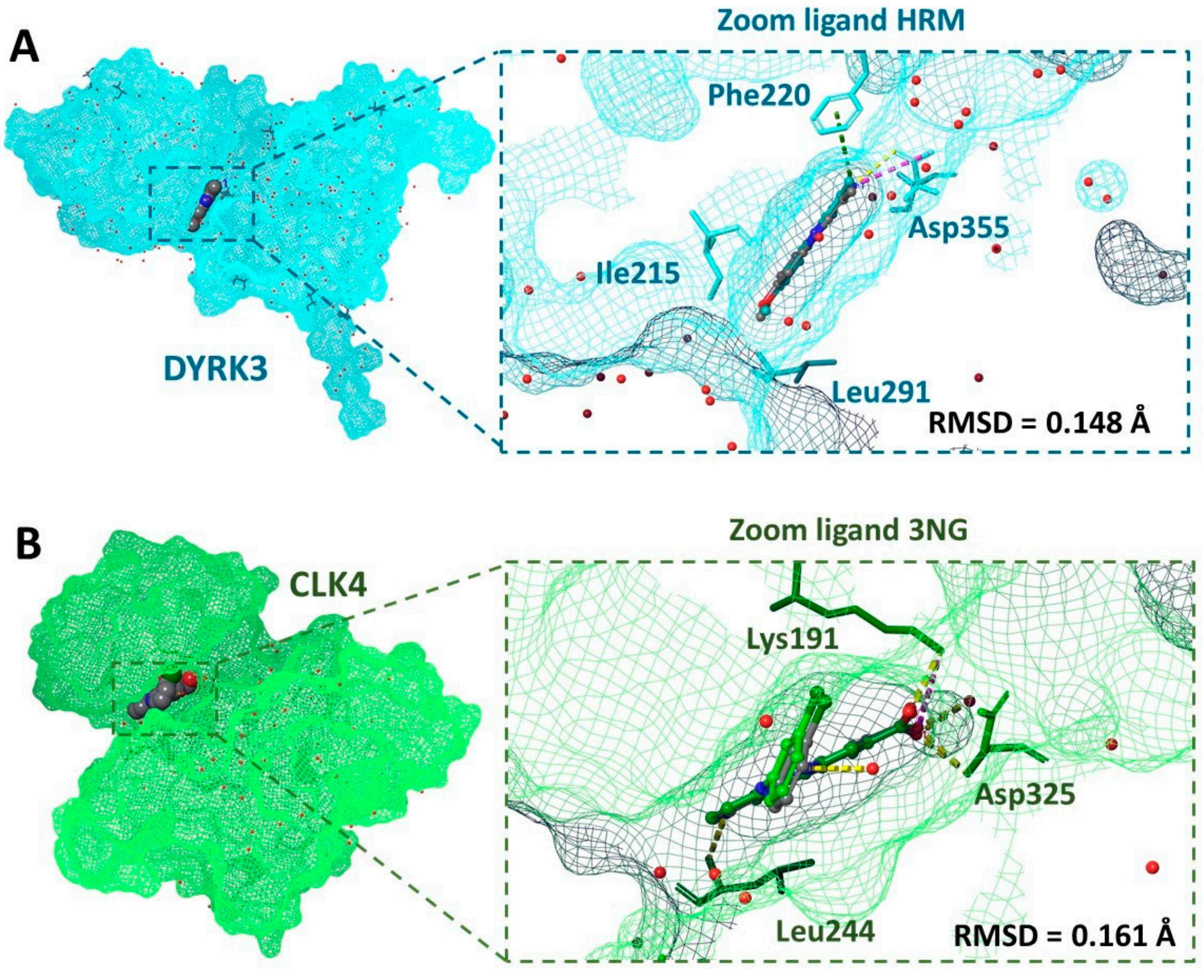

Figure S3. Visualization of the molecular re-docking procedure. *A*, compound **3NG** in the active site of CLK4. Geometric parameters of the green molecule correspond to the PDB ID: 6FYV; the position of the gray molecule was obtained by molecular docking. *B*, compound HRM in the active site of DYRK3. Geometric parameters of the green molecule correspond to the PDB ID: 5YH6; the position of the gray molecule was obtained by molecular docking. Hydrogen bonds are shown as yellow dotted lines. Salt bridges and π-cation stacking are rendered as purple and green dotted lines, respectively. Water molecules are represented as red balls. Gray molecules correspond to the geometrical parameters based on X-ray data. Blue and green molecules were obtained during the dockingprocedure.

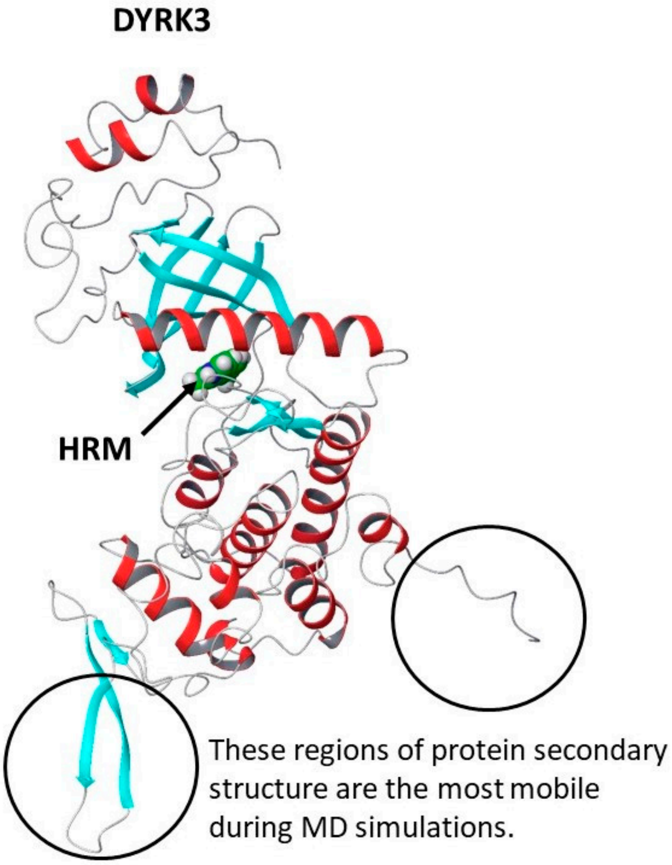

Figure S4. Secondary structures of DYRK3 and CLK4 and positions of reference inhibitors. Geometrical parameters of each structure were downloaded from Protein Data Bank. The structure of DYRK3 corresponds to ID: 6FYV, the structure of CLK4 to ID: 5YH6. Vibration of free loops in the DYRK3 structure is reflected in the fluctuation values (see Figure S3).

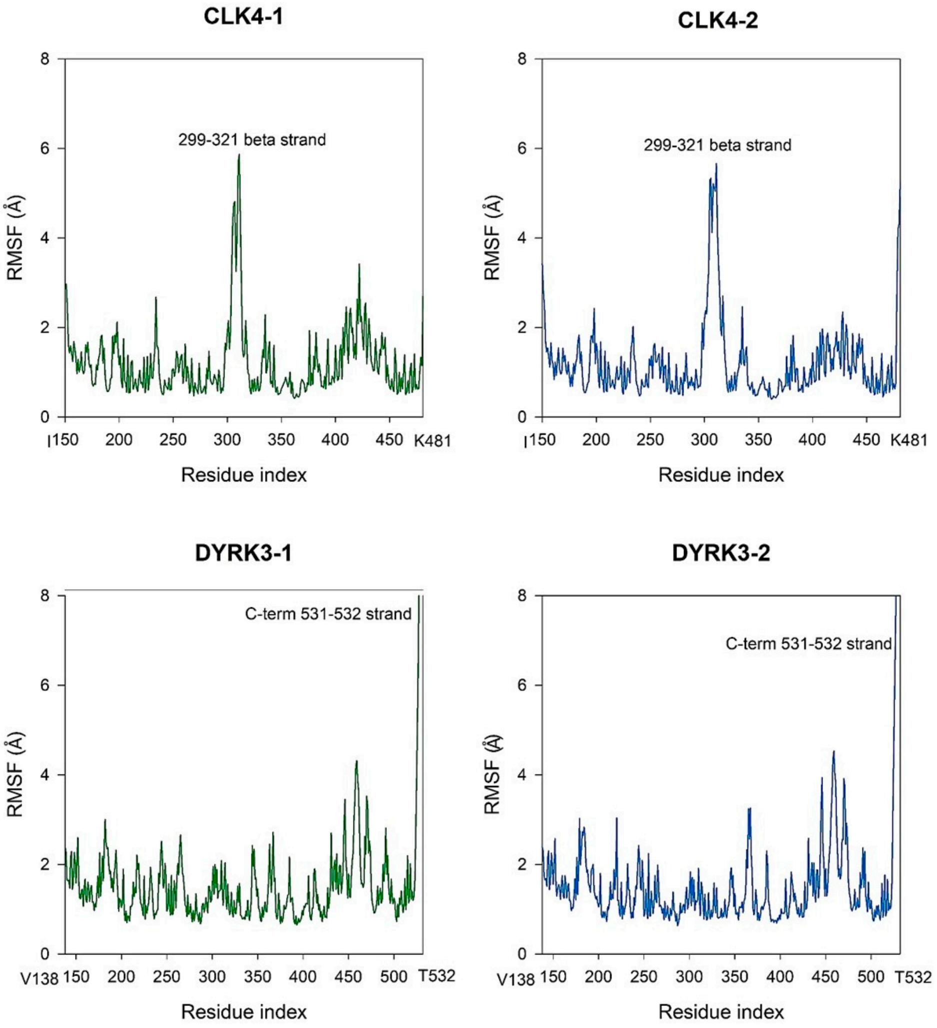

Figure S5. RMSF graphs of local changes of side chains of CLK4 and DYRK3 in complexes with **1** or **2**.

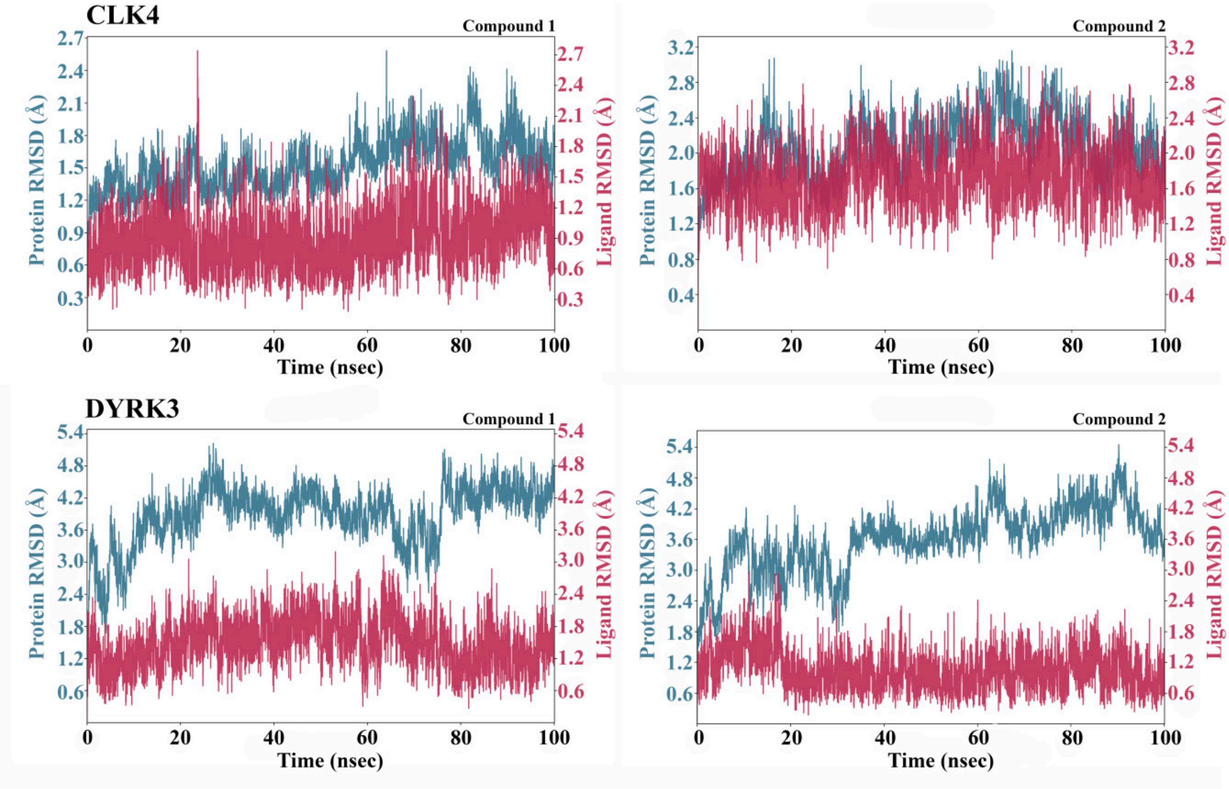

Figure S6. MD simulations. Shown are RMSD fluctuations of the ligand (dark red) and the target kinase (gray).

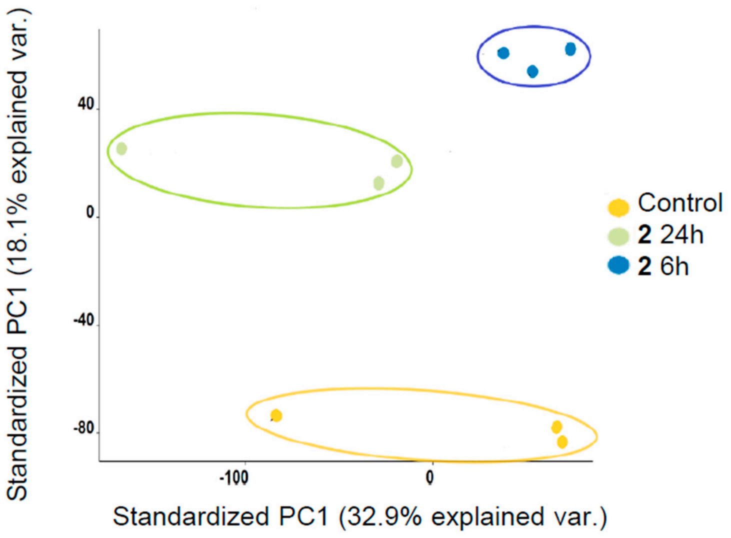

Figure S7. PCA analysis of RNAseq data. Experiments were performed in three biological replicates.

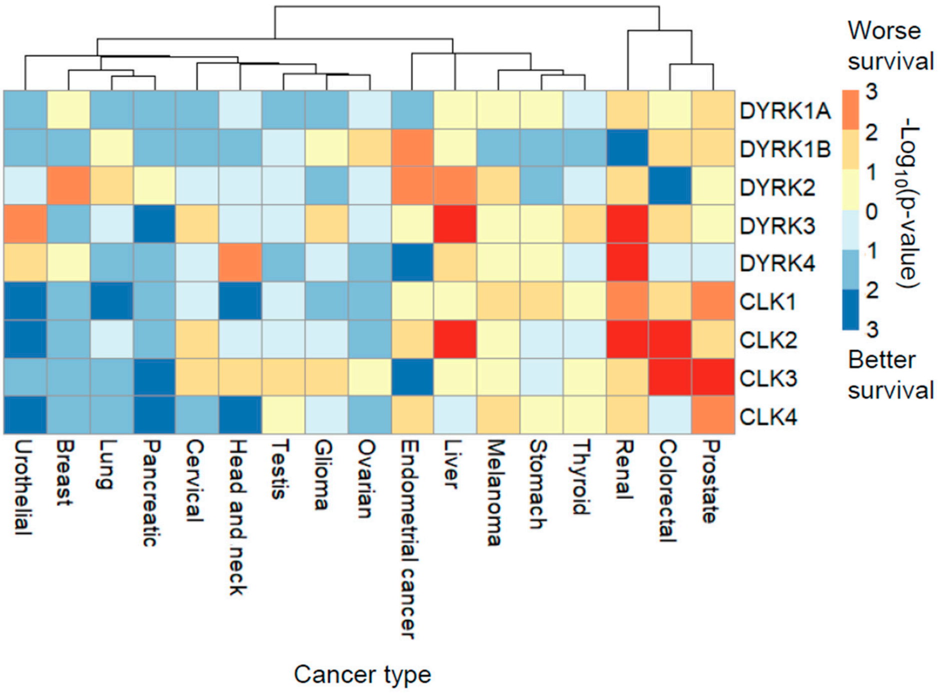

Figure S8. Correlation of the expression of DYRK/CLK family members with the survival of patients with different tumor types. The heat map was generated from TCGA database.

Table S1. Electron parameters of compounds **1** and **2**.

Compound	IP, eV	EA, eV	HOMO-LUMO gap	η	S	χ	ω
**1**	6.89	1.10	5.79	2.90	0.17	4.00	23.15
**2**	8.32	1.90	6.42	3.21	0.16	5.11	41.85

Table S2. Molecular docking of **1** and **2** into DYRK3 and CLK4 models.

Compound	IC_{50}, nM	Docking score	Pose	E_{model}	IFD score	H-bond	Other interactions	Clash	$\Delta G_{MM\text{-}GBSA}$, kcal/mol
**DYRK3**									
**HRM*** 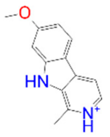	800 [9]	-7.55	9	-51.06	-834.94	Lys238	No	Glu253 Phe288 Asp355	-45.71
**1** 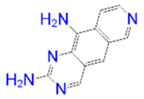	154[1]	-8.26	10	-52.86	-835.86	Leu291	No	Glu289 Val223 Ile354 Lys238	-42.06
**2** 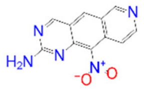	16[1]	-10.10	9	-67.23	-840.36	Leu291 Asp355	No	Glu289	-48.11
**CLK4**									
**3NG*** 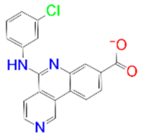	11[8]	-12.83	4	-119.57	-781.77	Leu244 Lys191 Asp325	Lys191-salt bridge	-	-46.60
**1** 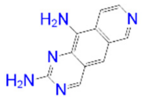	58700 [1]	-10.46	9	-68.91	-779.57	Leu244	No	Phe241 Glu242	-41.01
**2** 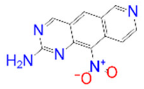	81 [1]	-10.18	8	-69.62	-779.87	Leu244	No	-	-44.15

*Values were obtained after the re-docking procedure.

Tables S3, S4 and **S5** are presented as separate Excel files.
